# General Remarks on Aliments Produced by the Different Classes of the Animal Kingdom, and Their Influence on the Human Body

**Published:** 1800-02

**Authors:** J. J. Virey


### *General Remarks on Aliments produced by the different Claſſes of the Animal Kingdom, and their Influence on the Human Body*; *by* J. J. Virey, *&c.*

[Continued from our laſt Number, pp. 65—70.]

THERE are few perſons unacquainted with the diſagreeable conſequences reſulting from the uſe of fiſh. This food is the cauſe of many cutaneous diſeaſes, ſuch as leproſy, itch, malignant ulcers, ſcurvy, and even dangerous diarrhœas. Mammiferous animals that feed on fiſh have a diſagreeable taſte; their fat is rancid, and their milk has a fetid ſmell. Several phyſicians have attributed certain endemic maladies to this aliment. Boate aſcribes the leproſy which afflicts the inhabitants of Ireland, who feed on fiſh, to this cauſe alone. Cleyer informs us, that ſalt fiſh occaſion elephantiaſis in the iſles of Sonda; and Prosper Alpinus aſſerts, that the leproſy of the Egyptians proceeds from fiſh. The liver, inteſtines, and ſkin of theſe

---

animals are often venomous. It is remarkable, that this food extends its action principally to the ſkin, which has a ſtrong ſympathy with the inteſtinal canal; and that the latter ſuffers dreadfully by the introduction of poiſon. The tetraodons, the diodons, and ſeveral other ſpecies, are the moſt venomous of the finny tribe. But I think that they are only ſo in conſequence of their aliment, which is compoſed of different zoophytes, as SONNERECK, FOSTER, and OSBECK have obſerved. The poiſon which ſome are ſuppoſed to poſſeſs in their prickly parts, as the *Trachinus draco*, LINN. the thornback, &c. does not actually exiſt. If the wounds occaſioned by theſe prickles, exhibit violent ſymptoms of inflammation, it is in conſequence of the laceration effected by theſe prickly parts, that are provided with ſmall crotchets. The Galvanic ſhock of cramp-fiſh, tetraodons, and others of an electric nature, are not dangerous except to their prey.

Whatever proportion of jelly fiſh may contain, they are not the moſt nutritive of animals. The taſte of their fleſh, like that of every other ſpecies of animal food, is leſs agreeable in the ſpawning ſeaſon, as it then emits a ſtrong ſcent. Fiſh of paſſage, as well as thoſe which live in muddy ponds, afford the moſt nutritive and delicious food; but others that move in mire, and whoſe reſpiration is more interrupted, have ſoft and flabby fleſh of a muddy taſte.

Let us now leave the great diviſion of animals, that have vertebræ and red blood, and deſcend to the inferior claſſes, which are by far the moſt numerous, but leſs beneficial to mankind. It appears that the utility of animals to man is in an inverſe ratio of their numbers, and this is one of the proofs that every thing was not created for man alone, as has been erroneouſly imagined.

We here meet with another ſyſtem of organization; and the nutritive ſubſtance preſents a new order of compoſition. The firſt claſs poſſeſs tough, glutinous juices, which are almoſt indigeſtible, and afford little nouriſhment. Theſe juices adhere to the tender fibres; they are a ſort of jelly which is eaſily decompoſed by a ſlow heat, and reſolved into a taſteleſs liquor that affords no aliment. In this ſtate, it is a viſcous, oily matter; but in the former, the ſubſtances are acrid and even corroſive: they exert their ſtimulating energy on the whole gaſtric ſyſtem, nay, frequently on the organs of generation.

Molluſques, whether naked or teſtaceous, afford in general an indigeſtible and ſlightly nouriſhing aliment, which is poſſeſſed of a certain mild cathartic, and ſometimes aphrodiſiacal property. They often have an inſipid and diſguſting taſte which riſes on the ſtomach, ſuch as the *aplyſie*, which poiſons the fleſh fleſh of thoſe fiſh that feed on it, and the *Mytilus lithophagus*, LINN. that yields a phoſphoric and rather acrid liquor. The fleſh of teſtaceous animals is not always uſed without danger, particularly at certain ſeaſons of the year. This poiſonous property is attributed to ſmall ſea-nettles, *Meduſæ*, LINN. and if acids act as an antidote againſt it, I have reaſon to believe, from a variety of obſervations, that acrid, ſtimulant, and aromatic ſubſtances increaſe its deleterious activity. Several *molluſques gymnodermes* afford an ardent liquor, ſimilar to the ink made from the cuttle and purple fiſh, as well as that of ſeveral other buccins and ſtrombes, &c. DIOGENES, the Cynic, having eaten a piece of their raw fleſh, died the ſame day. Broths made of ſome ſpecies of univalves have been ſucceſsfully employed in the cure of *ethiſies* (perhaps *phthiſis?*)

Cruſtaceous animals afford an agreeable and light but ſtimulant nutriment, like all the creatures included in this ſecond claſs of the animal kingdom: they often produce exanthematic eruptions, and a particular turgeſcence in the blood. They neither abound in albumen, nor jelly: the former of theſe humours appears to exiſt only in animals that have red blood.

The very numerous claſs of inſects afford but a ſmall proportion of aliment to man, and which is unwholeſome. Theſe animals often produce an extremely corroſive acid, which appeared to me uniformly the ſame; and I think that the bombic acid is ſo homogeneal with the formic, that there is no juſt ground for diſtinction. Inſects, when ſwallowed, act particularly on the ſecretory organs of the urine; and their influence alſo extends to the contiguous parts. The effects of certain inſects of the claſs *Coleoptera* are generally known, ſuch as the *Lytta veſicatoria*, FABR. *Meloes*, *Lampiris*, *Bupreſtis*, *Mylabris cichorii*, and ſeveral others. It is ſaid that the Hungarians ſwallow cantharides without danger, for the cure of a ſpecies of mania: ſeveral nations feeding upon inſects are alſo mentioned by writers, as well as ſome acridophages, ſuch as the Arabs and Moors. The quails of the Iſraelites were only a kind of locuſts, which produced inflammation in the throat. The continual uſe of honey alſo occaſions aphthous eruptions, and is ſaid to render the teeth carious, particularly in the ſouthern countries, where the heat accelerates the effect of all poiſons produced by organized bodies. There are, however, ſome particular idioſyncraſies in whom the internal uſe of inſects diſagrees: the learned ANNE SHURMAN is an inſtance of this peculiarity. I could quote a thouſand ſimilar examples. The women of Kamſchatka ſearch for ſpiders, and eat them with avidity, from an idea that ſuch food will render them prolific. We have lately diſcovered the anti-odontalgic property of feveral fpecies of the *curculio*, *carabus*, *chryfomela*, and *coccinella*; and that crickets poffefs the fame virtue as cantharides.

We fhall now confider the laft clafs of the animal kingdom, the moft diftant from mankind, that of the ftar-fifh and zoophytes. This clafs is lefs ufeful than thofe before-mentioned. The creatures it comprifes are formed of a fort of jelly that is entirely decompofed by heat. I will not infift, that they contain only a fmall quantity of nutriment; but I muft remark, that the greater part of them, far from being alimentary, are poifonous; and that their whole fubftance is compofed of a fort of naufeous and cauftic virus, which not only affects the ftomach, but extends its action over the whole furface of the body. If this deleterious quality (which increafes in proportion to the heat of the climate) does not affect the fifh feeding on it, whether of the mollufques or cruftaceous kind, yet the flefh of the latter eafily partakes of that poifonous property, and becomes deleterious to the more perfect animals, fuch as birds, quadrupeds, and man. It is unneceffary to mention numerous proofs of this fact, which might eafily be procured; as the mere contact of feveral of thefe animals has a cauftic effect, fufficient to poifon the prey on which they feed.

[ To be concluded in our next Number. ]

